# The introduction of ticagrelor is associated with lower rates of recurrent ischemic stroke after myocardial infarction

**DOI:** 10.1371/journal.pone.0216404

**Published:** 2019-05-06

**Authors:** Robin Henriksson, Fredrik Björklund, Thomas Mooe

**Affiliations:** Department of Public Health and Clinical Medicine, Östersund, Umeå University, Umeå, Sweden; University of Messina, ITALY

## Abstract

**Background:**

Previous ischemic stroke is a predictor of recurrent ischemic stroke after an acute myocardial infarction (AMI). Dual antiplatelet therapy, including a P2Y12-inhibitor, is important in secondary prevention after AMI. Ticagrelor, a P2Y12-inhibitor, is more potent than the commonly used clopidogrel. Here, we evaluated the impact of ticagrelor on the risk of ischemic stroke following AMI in patients with previous ischemic stroke.

**Methods:**

Data for patients with AMI that had a previous ischemic stroke were obtained from the Swedish Registry of Information and Knowledge about Swedish Heart Intensive Care Admissions. Patients were assigned to early and late cohorts, each covering a two-year time period before and after, respectively, the introduction of ticagrelor prescriptions (20 Dec 2011). Patients in the early cohort (n = 1633) were treated with clopidogrel (100%); those in the late cohort (n = 1642) were treated with either clopidogrel (66.3%) or ticagrelor (33.7%). We assessed the risk of ischemic stroke and intracranial bleeding over time with Kaplan-Meier analyses. We identified predictors of ischemic stroke with multivariable Cox regression analyses.

**Results:**

Of 3275 patients, 311 experienced ischemic stroke after AMI. Cumulative Kaplan-Meier incidence estimates of ischemic stroke within one year after AMI were 12.1% versus 8.6% for the early and late cohorts, respectively (p<0.01). Intracranial bleeding incidences (1.2% versus 1.5%) were similar between the two cohorts.

**Conclusions:**

Ticagrelor introduction was associated with a lower rate of ischemic stroke, with no increase in intracranial bleeding, in an AMI population with a history of ischemic stroke.

## Introduction

Cardiovascular disease is a leading cause of death and disability. It affects millions of people annually. Patients surviving an acute myocardial infarction (AMI) are at high risk for further vascular events. During the first year after AMI, the incidence of ischemic stroke is between 1.1–4.1%, depending on the patient selection[1–3]. Ischemic stroke after an AMI is associated with a major increase in morbidity and mortality[4]. A previous ischemic stroke is a major predictor of recurrent ischemic stroke following an AMI[3, 5, 6]. In the international REduction of Atherothrombosis for Continued Health (REACH) registry, 11.25% of patients with coronary artery disease had a history of ischemic stroke, which is often associated with significant co-morbidities[7]. Thus, these patients are routinely encountered in clinical practice and constitute an important high-risk population with a worse prognosis than the general AMI-population.

To reduce the risk of vascular complications after acute coronary syndrome, effective secondary prevention is fundamental. Due to fear of complications like bleeding, particularly intracranial bleeding, patients with a previous ischemic stroke are often treated conservatively, with regard to both interventions, such as percutaneous coronary intervention (PCI), and medications. Treatment with dual antiplatelet therapy (DAPT) is a cornerstone of secondary prevention after AMI[8, 9]. Until 2011 the standard DAPT in Sweden was the P2Y12 inhibitor, clopidogrel, in addition to aspirin. After the P2Y12-inhibitor, ticagrelor, was introduced in 2011, clinicians rapidly switched to ticagrelor as the preferred treatment option. Compared to clopidogrel, ticagrelor does not require metabolic activation, and its effect on platelet inhibition is more pronounced [10, 11]. The PLATelet inhibition and patient Outcomes (PLATO) trial clearly demonstrated that treatment with ticagrelor, compared to clopidogrel, reduced the rate of the composite primary endpoint of death from vascular causes, myocardial infarction, or stroke, but not stroke alone, in patients with ACS[1]. However, the PLATO-trial included, overall, healthier and younger individuals than the patients typically encountered in a real-life setting. The incidence of ischemic stroke in that trial was also lower than expected in a general AMI population. Currently, there is no clear evidence that ticagrelor is superior to clopidogrel in the prevention of ischemic stroke. Patients with a previous ischemic stroke have a particularly high risk of stroke after an AMI; for these patients, a potent anti-platelet treatment may be beneficial. Therefore, we aimed to determine whether the introduction of ticagrelor was associated with a lower rate of ischemic stroke following AMI in the high-risk subgroup of patients with a previous ischemic stroke.

## Methods

This was a registry-based observational study. Data for the study population were retrieved from The Swedish Web-based system for Enhancement and Development of Evidence-based care in Heart disease Evaluated According to Recommended Therapies (SWEDEHEART). SWEDEHEART is a national, quality registry, which includes the Registry of Information and Knowledge about Swedish Heart Intensive Care Admissions (RIKS-HIA). Since 2008, RIKS-HIA has received and stored data from all hospitals in Sweden with coronary care units. For each patient, over 100 variables are recorded at admission, during hospitalization, and at discharge. These data cover demographics, medical history, risk factors, biochemical markers, diagnoses, interventions, and medications. The validity of the data is examined annually, and there is 94–97% conformity between RIKS-HIA data and patient records[12].

AMI was defined according to the criteria established by the European Society of Cardiology/American College of Cardiology/American Heart Association[13]. The present analysis included all patients with AMI that had a history of previous ischemic stroke and were admitted between the 8^th^ of December 2009 and 31^st^ of December 2013 and discharged with either clopidogrel or ticagrelor. Patients were excluded when they were discharged with other P2Y12-inhibitors or without a P2Y12-inhibitor. Concomitant use of oral anticoagulants was not a reason for exclusion.

To identify patients with an ischemic stroke event during the study period and the date of death in applicable cases, the RIKS-HIA registry was combined with the National Patient Registry (NPR) and the Cause of death registry. The NPR includes the admission and discharge dates and the diagnoses at discharge for all hospital stays in Sweden. The International Classification of Diseases 10th Revision (I63 and I64) codes were used for recording cerebral infarction. The NPR has been validated, and a diagnosis of stroke had a positive predictive value of approximately 90%[14]. The registries included the Swedish population with no exclusion criteria.

The variable “smoking” was defined as smoking within the past month. “Atrial fibrillation” was defined as a previously identified atrial fibrillation or an atrial fibrillation during hospitalization. “Heart failure during hospitalization” was defined as the occurrence of pulmonary rales or treatment with intravenous diuretics during hospitalization. The estimated glomerular filtration rate (eGFR) was calculated with the Chronic Kidney Disease Epidemiology Collaboration equation, and all patients were assumed to be Caucasian[15].

Utilizing data from the Swedish prescribed drug registry, which is based on drug prescriptions dispensed from any pharmacy, we approximated treatment periods for clopidogrel and ticagrelor. The variable “Dispensed prescriptions” was categorized into four groups: treatment for three months (one prescription), six months (two prescriptions), nine months (three prescriptions), and twelve months or more (at least four prescriptions). Patients were excluded from the analysis of treatment length and considered as missing data when, for any reason, they switched antiplatelet therapy during the course of the study. The RIKS-HIA registry was approved by the National Board of Health and Welfare and the Swedish Data Inspection Board. The Regional Ethics Committee in Stockholm approved the merging of registries.

### Statistical analysis

We compared two different time periods, before and after the introduction of ticagrelor, instead of directly comparing the effects of two drugs. This model was chosen to avoid a selection bias, given that ticagrelor was the preferred treatment for patients at low-risk, after it was introduced into routine care. The first prescription of ticagrelor was used as the time point (20^th^ December 2011) between two time periods of similar length. The early period lasted from the 8^th^ of December 2009 to the 19^th^ of December 2011; the late period lasted from the 20^th^ of December 2011 to the 31^st^ of December 2013. Patients in the early cohort received clopidogrel exclusively, but patients in the late cohort received either clopidogrel or ticagrelor. Statistical results are presented as percentages for categorical variables and as the median with 25^th^ and 75^th^ percentiles for continuous variables. We compared the early and late cohorts for differences in baseline characteristics and differences in medications at admittance and at discharge. The Mann-Whitney U-test was used to evaluate continuous variables, and the Pearson Chi-square test was used to evaluate categorical variables. Kaplan-Meier analysis was used to estimate the incidence of ischemic stroke in the early and late cohorts and to estimate the incidence of intracranial bleeding in the two cohorts. Univariable and multivariable Cox regression analyses were performed to identify predictors of ischemic stroke. Baseline variables (clinical characteristics and treatments) that predicted risk in the univariable Cox analysis (p <0.1) were included in the multivariable Cox regression analysis. The final multivariable Cox regression analysis was based on stepwise exclusion of non-significant variables. We used scaled Schoenfeld residuals to verify that the proportional hazards assumption was not violated. Results from the Cox regression analyses are presented as hazard ratios (HR) with a 95% confidence interval (CI). For all statistical analyses, a P-value <0.05 was considered significant. All analyses were performed with IBM SPSS v 23.

## Results

The study included 3275 patients with AMI that had experienced a prior ischemic stroke. Of these, 1633 patients were in the early cohort, exclusively treated with clopidogrel, and 1642 patients were in the late cohort, treated with either clopidogrel (n = 1089, 66.3%) or ticagrelor (n = 553, 33.7%).

### Baseline characteristics

The baseline characteristics are shown in Table 1. Overall, the patient characteristics were similar between cohorts; however, some significant differences emerged. In the late cohort, the mean age was slightly older, and hypertension was significantly more common than in the early cohort. Additionally, during hospitalization, the rate of PCI treatment was significantly higher, and the rate of thrombolysis was significantly lower in the late cohort compared to the early cohort.

**Table 1:** Baseline characteristics of the early vs. late cohort.

Variable	Early cohort (n = 1633)	Late cohort (n = 1642)	P-value
Age, y, median, (IQR*)	78 (70.5–83)	78 (71–84)	0.05
Women	35.3	38	0.11
Smoking	14.3	14	0.83
Diabetes	36.7	36.1	0.74
STEMI ^†^	28.5	25.9	0.01
Hypertension	81	83.7	0.04
Atrial fibrillation	26.2	27.4	0.44
Heart failure during hospitalization	34.6	32.1	0.13
Previous MI ^‡^	18.7	16.9	0.18
Previous intra-cerebral hemorrhage	3.4	4.4	0.13
Previous dialysis	0.8	1.2	0.23
Previous PAD ^§^	12.4	11.8	0.55
Thrombolysis during hospitalization	1	0.4	0.02
PCI ^| |^ during hospitalization	49.9	55.7	0.001
CABG ^#^ during hospitalization	0.9	1.4	0.14
eGFR**, median (IQR)	62.8 (45.1–79.6)	62.1 (45–79.3)	0.52

Values represent percentages (%), unless otherwise indicated.

*IQR: interquartile range

† STEMI: ST-elevation myocardial infarction

‡ MI: myocardial infarction

§ PAD: Peripheral artery disease

| | PCI: Percutaneous coronary intervention

# CABG: Coronary artery bypass graft surgery

** eGFR: Estimated glomerular filtration rate.

### Medication

Overall, patients received similar use of cardiovascular medications at admission, except the late cohort reported a significantly lower use of aspirin compared to the early cohort (65.6% vs 69.5% p = 0.02). There were no significant difference in the use of ACE-inhibitors/angiotensin receptor blocker, statins, oral anticoagulants, beta blockers, calcium inhibitors or diuretics.

At discharge in the late cohort, significantly more patients were treated with oral anticoagulants (9% vs 11.6% p = 0.01), and fewer patients were treated with aspirin (92.8% vs 88.3% p = <0.001), beta blockers (88.4% vs 85.6% p = 0.03), and diuretics (47.5% vs 42.3% p = <0.01), compared to the early cohort. The cohorts included similar proportions of patients that received ACE-inhibitors/angiotensin receptor blockers, statins, and calcium channel inhibitors.

### Incidence of ischemic stroke and intracranial bleeding

**Fig 1:**
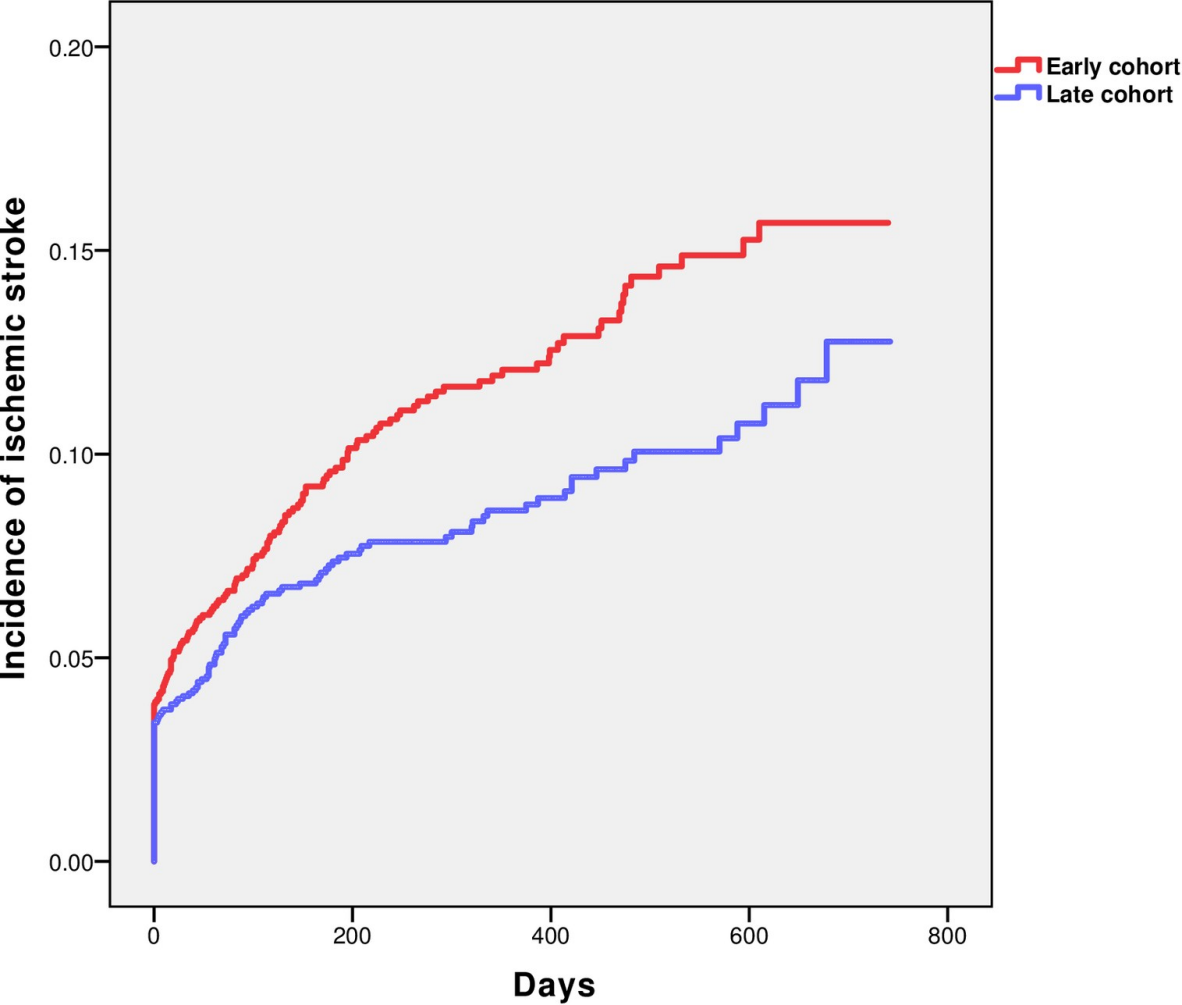
Incidence of ischemic stroke during the study period among patients with AMI with prior ischemic stroke.

The Kaplan-Meier analysis showed the incidence of ischemic stroke (Fig 1). Out of 3275 patients, 311 experienced ischemic stroke during the study period. Cumulative Kaplan-Meier incidence estimates of ischemic stroke after one year were 12.1% (n = 162) in the early cohort and 8.6% (n = 120) in the late cohort (p<0.01, based on log-rank test). There was no significant difference between the estimated incidences of intracranial bleeding in the two cohorts; 1.2% (n = 14) versus 1.5% (n = 17).

### Predictors of ischemic stroke

In the univariable Cox regression analysis atrial fibrillation predicted a higher risk of ischemic stroke (HR 1.41 (1.12–1.79) p<0.01). A lower risk of ischemic stroke was predicted by a PCI during hospitalization (HR 0.7 (0.56–0.88) p<0.01), treatment with statins at discharge (HR 0.71 (0.54–0.93) p = 0.02), and belonging to the late cohort (HR 0.73 (0.58–0.91) p<0.01). The final multivariable Cox regression analysis included 3272 patients (Table 2). Atrial fibrillation predicted an increased risk of ischemic stroke, and a PCI during hospitalization predicted a reduced risk of ischemic stroke. Membership in the late cohort was associated with a 26% reduction in the HR for ischemic stroke (p <0.01).

**Table 2:** Multivariable predictors of ischemic stroke.

Name of predictor	HR (95% CI)	P-value
Atrial fibrillation	1.39 (1.1–1.77)	<0.01
PCI* during hospitalization	0.73 (0.58–0.91)	<0.01
Belonging to the late cohort	0.74 (0.59–0.93)	<0.01

*PCI: Percutaneous coronary intervention

### Prescription patterns for clopidogrel and ticagrelor

The number of prescriptions dispensed from pharmacies for clopidogrel and ticagrelor in the early and the late cohorts where calculated. There was a trend towards more dispensed prescriptions in the late cohort, which translated into longer treatment durations. Four or more prescriptions were dispensed to 38.2% of patients in the early cohort. In the late cohort, four or more prescriptions were dispensed to 42.2% of patients that received clopidogrel and 57.9% of patients that received ticagrelor. Switching between treatments during the study period resulted in missing data; this problem was most notable among patients treated with clopidogrel in the late cohort (21.1%).

## Discussion

The present analyses were based on data from a fairly unselected population of patients with AMI that had previously experienced an ischemic stroke. The 1-year risk of recurrent ischemic stroke was markedly lower in the late cohort (8.6%) than in the early cohort (12.1%). The multivariable adjusted analysis showed that patients in the late cohort had a 26% lower hazard ratio for ischemic stroke than patients in the early cohort. The risk of intracranial bleeding was similar in the two cohorts.

The median coverage of the RIKS-HIA registry was greater than 90% of all patients with a diagnosis of myocardial infarction in Swedish hospitals[16]. In the present study, this high coverage was reflected by cohorts with a high median age (78 years) and a high prevalence of different co-morbidities. Therefore, the external validity of our results should be high, at least in Caucasian populations.

We currently lack clear evidence of the efficacy of the P2Y12-inhibitor, ticagrelor, for the prevention of ischemic stroke. The role of ticagrelor in secondary prevention after acute coronary syndrome was established by the PLATO-trial. There, ticagrelor reduced the rate of the composite primary end-point of vascular death, myocardial infarction, or stroke, compared to clopidogrel[1]. However, no difference was found in the incidence of ischemic stroke between groups treated with clopidogrel or ticagrelor (1.1% vs. 1.1%). The Prevention of Cardiovascular Events in Patients with Prior Heart Attack Using Ticagrelor Compared to Placebo on a Background of Aspirin–Thrombolysis in Myocardial Infarction 54 (PEGASUS-TIMI 54) trial showed that long-term use of ticagrelor on top of aspirin compared to aspirin alone reduced cardiovascular death, myocardial infarction, or stroke, when ticagrelor treatment was started more than 1 year after a myocardial infarction. The three-year Kaplan-Meier estimate of ischemic stroke was reduced in the group that received 60 mg of ticagrelor twice daily compared to placebo, but the difference (1.65% vs. 1.28%) did not achieve statistical significance (p = 0.06)[17]. In the Acute Stroke or Transient Ischaemic Attack Treated with Aspirin or Ticagrelor and Patient Outcomes (SOCRATES) trial, ticagrelor, compared to aspirin, did not reduce the composite primary endpoint of stroke, myocardial infarction, or death at 90 days after acute stroke or transient ischemic attack[18]. However, the secondary endpoint of ischemic stroke was significantly reduced in patients treated with ticagrelor (5.8%) compared to patients treated with aspirin (6.7%; p = 0.046). However, due to the pre-specified hierarchical testing plan, that result was deemed insignificant.

Despite those findings, there is a small, but growing body of evidence that has suggested that ticagrelor might play a part in the prevention of ischemic stroke. Studies have demonstrated reductions in ischemic stroke when ticagrelor treatment was evaluated in patients with a history of established atherosclerotic disease. In a pre-specified exploratory analysis of patients included in SOCRATES, patients with ipsilateral extracranial or intracranial stenosis experienced a 6.4% recurrence rate of ischemic stroke when treated with ticagrelor, compared to 8.5% when treated with aspirin (p = 0.02)[19]. In a substudy of the PEGASUS trial, the incidence of stroke was investigated in the group that received the approved, 60 mg twice-daily dose of ticagrelor. Compared to aspirin, ticagrelor significantly reduced the risk of stroke (HR 0.75, p = 0.034), and this reduction was driven by a reduction in ischemic stroke[20].

In the present study, we found a remarkably high rate of recurrent ischemic stroke. A much lower risk of stroke (overall) was found in a PLATO substudy, which examined outcomes in 1152 patients with a history of stroke or transient ischemic attack. The Kaplan-Meier stroke rate was 3.4% (n = 36) at 360 days. Those authors found that their results were consistent with the overall PLATO results, and that ticagrelor had no additional effect on stroke prevention[21]. The low stroke incidence, compared to our findings, could be explained by the overall younger population with fewer co-morbidities compared to our AMI-population. Our results showed that patients with AMI that had experienced a previous ischemic stroke had a one-year risk of recurrent ischemic stroke similar to that of patients hospitalized with an acute stroke[22]. Our late cohort had a considerably lower one-year incidence of recurrent stroke than the early cohort; the absolute difference was 3.5%. A difference of this size could not be explained by the observed group differences at baseline. The two cohorts showed only minor differences in the most important known predictors of stroke: age, hypertension, atrial fibrillation, diabetes, and congestive heart failure.

An oral anticoagulant (OAC) was more frequently used in the late cohort than in the early cohort (absolute difference 2.6%). However, OAC use did not predict a reduced risk of ischemic stroke when assessed as a variable in the univariable Cox regression analysis.

In the adjusted analysis, we identified two factors that predicted reduced risk of recurrent ischemic stroke. The first factor was a PCI, which has been associated with a reduced risk of stroke in other AMI populations[3, 6, 23]. It has been hypothesized that the preventive effect of a PCI may be explained by the low rate of myocardial injury, which reduces the potential for inflammatory reactions and changes in platelet reactivity[23, 24]. The second factor was belonging to the late cohort. The association between belonging to the late cohort and the lower rates of stroke was similar to the association observed for the PCI.

Our study population differed markedly from populations studied in randomized trials with ticagrelor. In evaluations of stroke risk, the study population may be of major importance. A high-risk population, characterized by a high mean age, extensive co-morbidities, the presence of inflammation, and a high degree of platelet activation, may derive particularly high benefit from treatment with a potent antiplatelet drug, such as ticagrelor. Therefore, the increased use of ticagrelor in the late cohort might at least partly explain the association of the late cohort with a lower rate of stroke. This hypothesis was supported by findings in the previously described substudies[19, 20].

Increased platelet inhibition is associated with increases in bleeding complications. In the present study, the higher frequency of ticagrelor treatment in the late cohort led to increased platelet inhibition compared to the early cohort, which received clopidogrel exclusively. However, we did not observe a significant difference between groups in the most serious bleeding complication–intracranial bleeding. This suggested that ticagrelor may be a valid treatment option in this high-risk population. Clearly, there is an urgent need for an effective treatment to reduce the risk of recurrent stroke in patients with AMI that have experienced a previous ischemic stroke. Our results demonstrated the need for a randomized trial on ticagrelor.

### Limitations

When using registry data, there is an inherent risk that confounding factors might be missed, and this might alter the results. For example, we may not have accounted for all changes in treatment routines over the study period that may have been important in our analyses. However, we limited the extent of selection bias by comparing cohorts before and after the introduction of ticagrelor, rather than performing a direct comparison between ticagrelor and clopidogrel treatments. Due to the inherent limitations of a registry-based study, and the overall indirect design of our comparisons, we could not obtain a precise estimate of the actual treatment effect. In addition, the analysis of the treatment period as a predictor of outcome was weakened, due to missing data. Moreover, we could not estimate adherence to treatments prescribed at discharge; therefore, we assumed all patients ingested prescribed drugs regularly. Although there was no significant difference in the incidence of intracranial bleeding between the two cohorts, more studies are warranted to obtain more detailed assessments of bleeding outcomes in this high-risk population.

### Conclusion

This study showed that, in an AMI population with a history of ischemic stroke, the introduction of ticagrelor was associated with a lower rate of ischemic stroke and no increase in intracranial bleeding.
